# Multiple myeloma with acute light chain cast nephropathy

**DOI:** 10.1038/s41408-023-00806-w

**Published:** 2023-03-29

**Authors:** Nelson Leung, S. Vincent Rajkumar

**Affiliations:** 1 Division of Hematology, Mayo Clinic, Rochester, MN USA; 2 Division of Nephrology and Hypertension, Mayo Clinic, Rochester, MN USA

**Keywords:** Myeloma, Therapeutics

## Abstract

Light chain cast nephropathy (LCCN) is a leading cause of acute kidney injury (AKI) in patients with multiple myeloma (MM) and is now defined as a myeloma defining event. While the long-term prognosis has improved with novel agents, short-term mortality remains significantly higher in patients with LCCN especially if the renal failure is not reversed. Recovery of renal function requires a rapid and significant reduction of the involved serum free light chain. Therefore, proper treatment of these patients is of the utmost importance. In this paper, we provide an algorithm for treatment of MM patients who present with biopsy-proven LCCN or in those where other causes of AKI have been ruled out. The algorithm is based on data from randomized trial whenever possible. When trial data is not available, our recommendations is based on non-randomized data and expert opinions on best practices. We recommend that all patients should enroll in a clinical trial if available prior to resorting to the treatment algorithm we outlined.

## Introduction

Acute kidney injury (AKI) caused by light chain cast nephropathy (LCCN) is one of the major complications from multiple myeloma (MM). It is most commonly seen at initial MM diagnosis but can also develop later in the course of disease during relapse. The incidence AKI at diagnosis is 16–31% when measured by serum creatinine (Scr) concentration >1.4 mg/dl, and 16–22% when defined using Scr >2 mg/dl [1, 2]. By estimated glomerular filtration rate (eGFR), 36–45% have an eGFR < 60 ml/min/1.73 m^2^, while 12–17% have an eGFR <30 ml/min/1.73 m^2^ [2–4]. A large French study of 1038 patients with MM found that ~25% met the 2014 International Myeloma Working Group (IMWG) renal impairment criterion (Scr > 2 mg/dl or eGFR < 40 ml/min/1.73 m^2^), with 12.9% requiring dialysis [5]. Other have reported dialysis dependence in 6–8% of patients during the clinical course of MM [4, 6].

Of the four myeloma defining events (MDEs) (hyper**C**alcemia, **R**enal impairment, **A**nemia and **B**one lytic lesions), renal impairment imposes the greatest impact on overall survival (OS) even after adjusting for other cofactors and comorbidities [2–6]. This was especially evident in the alkylator era [6, 7]. Although this effect is diminished by novel agents particularly bortezomib in clinical trial settings [8, 9], real world data still reflect that AKI at the time of myeloma diagnosis imposes a negative impact on mortality particularly in the first 6 months of therapy [4, 5, 10]. Fortunately, recovery of kidney function reverses the negative impact on OS [5–7, 10]. But while long-term survival has improved, short-term mortality remains higher in patients without kidney recovery regardless of the treatment regimen [8].

## Mechanisms of renal impairment

In 2014, the IMWG clarified that only AKI secondary to LCCN qualifies as a MDE [11]. LCCN occurs when the overproduced monoclonal free light chain (FLC) by the myeloma cells interacts with the Tamm Horsfall protein in the loop of Henle to form light chain casts [12]. The light chain casts obstruct the tubules causing rupture which induces an immune response further injuring the tubules [13]. Injury is also mediated by hydrogen peroxide produced by the FLC and FLC activation of the Nuclear factor kappa-light-chain-enhancer of activated B cells (NF-κB) and Apoptosis signal-regulating kinase 1 (ASK1) or Janus kinases, signal transducer and activator of transcription proteins (STATs) pathway [13–15]. These pathways induce apoptosis, inflammation and promote tubulointerstitial fibrosis that further the damage beyond tubular objection.

Serum FLC concentrations are predictive of the development of AKI and its recovery. AKI is rare when serum FLC concentration is <50 mg/dl but increases significantly when the concentration exceeds 80–200 mg/dl [16, 17]. A high serum FLC concentration alone may not be sufficient as a high urinary FLC excretion appears to be necessary for AKI to occur [7, 18, 19]. Conversely, a rapid reduction of serum FLC concentration is the key to reversing the kidney injury. A minimum reduction of 50–60% of serum FLC has been found to be associated with renal recovery in LCCN [20, 21]. One study finds that fewer patients recovered kidney function with the same degree of FLC reduction achieved at day 21 as compared to day 12 [22]. Finally, a serum FLC concentration of <50 mg/dl at the end of cycle 1 of chemotherapy is associated with better renal recovery in a recent clinical trial using a high cutoff (HCO) dialyzer [23].

## Diagnosis

Renal impairment in MM is currently defined by an eGFR of <40 ml/min/1.73 m^2^ or a Scr >2 mg/dl [11]. For diagnostic and management purposes, the etiology of AKI in MM must be established. Only LCCN qualifies as a MDE, and its management is different from the management needs for AKI due to other causes. Thus, in patients with MM presenting with AKI, other causes of renal failure such as dehydration, hypercalcemia, drug-induced nephritis, unrelated diabetes or hypertension, and other monoclonal gammopathy related renal pathology must all be excluded. There are a number of renal disorders than are caused by monoclonal proteins besides LCCN that are collectively incorporated in the diagnostic umbrella now termed monoclonal gammopathy of renal significance (MGRS) [24]. Kidney biopsy is the gold standard for differentiating between LCCN, MGRS lesions, and other unrelated causes of AKI [25]. Unfortunately, a kidney biopsy is not always feasible for various reasons, especially in the acute setting. Once dehydration, hypercalcemia, and other causes of AKI are excluded based on clinical presentation, the main differential is between LCCN and MGRS renal lesions. Since most MGRS kidney lesions affect the glomeruli, they cause a high degree of albuminuria. In contrast, LCCN is associated with mainly Bence Jones proteinuria often with <10% albuminuria [26]. Therefore, in patients with >1 g/d proteinuria with <10% albuminuria, and a serum FLC concentration >150 mg/dl, the probability of LCCN is high enough that a kidney biopsy can be omitted [7, 26, 27]. On the other hand, patients with high degree of albuminuria or lower serum FLC, or where there is any uncertainty about the etiology of AKI should undergo a kidney biopsy.

## Current frontline treatment options

### Proteasome inhibitors (Table 1)

Bortezomib is a reversible proteasome inhibitor that is not renally cleared nor nephrotoxic [28]. In the VISTA trial, the addition of bortezomib (V) to melphalan and prednisone (MP) significantly increased the overall response rate (ORR) from 46 to 68%, and the complete response (CR) rate from 5 to 31%, respectively in patients with eGFR < 50 ml/min/1.73 m^2^ [9]. Renal recovery rate in those with an eGFR < 30 ml/min/1.73 m^2^ was 37% in the VMP arm vs 7% in the MP treated patients. Bortezomib was combined with doxorubicin and dexamethasone (BDD) in a phase II study in both newly diagnosed (NDMM) and relapsed myeloma patients with a median eGFR of 20.5 (3.7–49.9) ml/min. BDD produced an ORR of 72%, with a very good partial response (VGPR) or better rate of 52% [29]. Improvement in eGFR correlated with the depth of hematologic response (HR) with the median posttreatment eGFR of 59.6 ml/min, 38.9 ml/min and 16.8 ml/min in patients who achieved >VGPR, partial response (PR) or minimal response (MR), and stable disease or less, respectively. Median progression free survival (PFS) of this study was 12.1 months and OS had not been reached after a median follow-up of 22.4 months. In the phase III Hovon-65/HMMG-HD4 trial, BDD was compared to vincristine Adriamycin and dexamethasone (VAD) induction followed by autologous stem cell transplant (ASCT) followed by thalidomide maintenance in the VAD group and bortezomib maintenance in the BDD group [30]. Despite a significantly higher prevalence of high risk cytogenetics [del17p and t(4;14)] in the patient with Scr >2 mg/dl, the OS of patients treated with BDD was similar to those with baseline creatinine ≤2 mg/dl [8]. BDD achieved a significantly higher ORR and >VGPR rate (75% and 33%, respectively) in the patients with a baseline creatinine >2 mg/dl as compared to VAD (36% and 9%, respectively). Patients with a baseline creatinine ≤2 mg/dl had similar OS regardless of treatment but those with Scr >2 mg/dl treated with VAD had an inferior OS. Overall renal response was 81% (58% renal CR) in the BDD treated patients vs 63% (43% renal CR) in the VAD treated patients. A randomized trial comparing bortezomib plus dexamethasone (BD) to bortezomib, cyclophosphamide, dexamethasone (CBD) found similar HR (78.3% in BD vs 77.2% in CBD, *p* = 1.00) and >VGPR rate (39.1% in BD and 51.1% in CBD, *p* = 0.14) in NDMM with renal impairment not requiring dialysis at 3 months [31]. There was no difference in the overall renal response (44.6% in BD vs 51.1% in CBD, *p* = 0.46) but recovery of patients with AKI stage 3 (creatinine increased >3 times of baseline) trended toward CBD therapy (23.2% in BD vs 46.7% in CBD, *p* = 0.07).

**Table 1:** Multiple myeloma trials with renal impaired patients.

Trial	Regimen	Patients	Inclusion criteria	Hematologic and Renal Outcomes	Toxicity
Phase III VISTA	MP vs VMP	NDMM 450 with CrCl >50 ml/min 227 CrCl ≤ 50 ml/min	Scr <2 mg/dl	HR in patients with CrCl <50 ml/min ORR: MP = 46% vs VMP = 68% CR: MP = 5% vs VMP = 31% Renal response Overall: MP = 34% vs VMP = 44% CrCl < 30 ml/min: MP = 7% vs VMP = 37% CrCl 30 - < 50 ml/min: MP = 39% vs VMP = 46%	Grade 4 hematologic AEs (42%) and SAEs (63%) were higher in patients with CrCl ≤ 30 ml/min but discontinuation rates were similar. Patients who achieved reversal of RI had less grade 5 AEs (8% vs 15%), SAEs (43% vs 60%) and discontinuation (6% vs 24%) than patients without reversible RI.
Phase II study Ludwig et al.	BDD	68 NDMM and RMM	eGFR < 50 ml/min	Hematologic response Renal response CR/nCR = 38% CR^renal^ = 31% VGPR = 15% PR^renal^ = 7% PR = 13% MR^renal^ = 24% MR = 6%	Grade 3/4 AEs Anemia - 50% Neutropenia - 14.7% Thrombocytopenia -14.7% Infections: Grade 4 - 19.1%, Grade 3 - 48.5%
Phase III Hovon-65/ HMMG-HD4	PAD/ASCT/Bortezomib maintenance vs VAD/ASCT/thalidomide maintenance	NDMM 81 with Scr ≥ 2 mg/dl 746 with <2 mg/dl	No exclusion for kidney function	HR in patients with Scr > 2 mg/dl ORR: PAD 75% vs VAD 36% >VGPR: PAD 33% vs VAD 9% Renal response ORR: PAD = 81% vs VAD = 63% CR^renal^: PAD = 58% vs VAD = 43% PR^renal^: PAD = 0% vs VAD = 3% MR^renal^: PAD = 23% vs VAD = 17%	Grade 4 AEs Anemia: VAD (7%) vs PAD (8%) Neutropenia: VAD (1%) vs PAD (3%) Thrombocytopenia: VAD (5%) vs PAD (10%)* Infections: VAD (21%) vs PAD (26%) Nonhematologic AE GI symptoms: VAD (7%) vs PAD (11%)^#^ PN: VAD (10%) vs PAD (24%)^#^ * = *p* < 0.01, ^#^ = *p* < 0.001
Phase III MYRE	BD: bortezomib 1.3 mg/m^2^, dex 20 mg Days (1,2,4,5, 8,9,11,12) cycle 1, cycle 2 is 28 days for age >70 years vs CBD: BD + iv CTX 750 mg/m^2^ Day 1	184 NDMM with LCCN	Scr > 1.92 mg/dl or eGFR < 40 ml/min/1.73m2	Hematologic response at 3 months ORR: BD = 78.3% vs CBD = 77.2% CR/VGPR: BD 39.1% vs CBD = 51.1% Renal response at 3 months ORR: BD = 44.6% vs CBD = 51.1%	Serious AEs BD – 32.6% vs CBD – 43.5% Grade ≥ 3 Cytopenia: BD (5.4%) vs CBD (9.8%) Sepsis/pneumonia: BD (2.2%) vs CBD (5.4%) None were statistically significant
Tosi et al.	T or TD Thalidomide 100–400 mg daily Dex- 40 mg days 1–4	20 RRMM	Scr > 1.5 mg/dl or CrCl < 60 ml/min	Hematologic response at 3 months ORR: 75% PR: 45% Renal response (Scr < 1.5 mg/dl) ORR: 80%	Constipation – 25% Lethargy – 25% Leukopenia – 5% 50% of patients on 400 mg dose could not tolerate the dose due to Grade ≥ 2 toxicity.
Analysis of the phase III MM-009 and MM-010 Dimopoulos et al.	Lenalidomide 25 mg days 1–21 Dex 40 mg Days 1–4, 9–12, 17–21	341 RRMM	Scr ≥ 2.0 mg/dl	Hematologic response CR: mild RI 16%, mod RI 16%. sRI 6% VGPR: mild RI 19%, mod RI 11%, sRI 31% PR: mild RI 30%, mod RI 29%, sRI 13% Improvement of renal function by 1 level was experienced by 72%	Neutropenia: 32–48%. Thrombocytopenia: 38% severe RI, 22% moderate RI and 9% mild/no RI. Anemia: 44% severe RI, 21% moderate RI, 5% mild/no RI Pneumonia: 25% severe RI, 9% moderate RI, 7% mild/no RI. Dehydration: 13% severe RI, 2% moderate RI and 0.8% mild/no RI
Phase II study Ludwig et al.	Lenalidomide dosed based on CrCl Dex 40 mg Days 1–4, 9–12, 17–21 cycle 1 and weekly afterwards	35 NDMM RRMM LCCN with 15 biopsy confirmed	Scr > 2 mg/dl or CrCl < 50 ml/min	Hematologic and Renal response CR: 20% VGPR: 8% PR: 40% CR^renal^: 14.2% PR^renal^: 11.4% MR^renal^: 20%	Grade 3/4 toxicities Anemia: 43% Thrombocytopenia: 23% Leukopenia: 12% Neutropenia: 15% 3 patients withdrew due to AE and 4 deaths during the first 2 cycles
Phase II study Bridoux et al.	Lenalidomide (dose based on kidney function) Dex 40 mg weekly	38 RRMM	No Scr cutoff but renal function needed to be stable	Hematologic response ORR: 76% CR: 0% VGPR: 26% PR: 50% ≥50 ml/min: 75% vs <50 ml/min: 78%	Nonserious AEs Thrombocytopenia: normal (40%) vs sRI/HD (63.6%) Leukopenia: normal (20%) vs sRI/HD (36.4%) Anemia: normal (0%) vs sRI/HD (27.3%) Fatigue: normal (20%) vs sRI/HD (45.5%)
PrE1003	Lenalidomide dose escalation Dex 40 mg weekly	63 RRMM		Hematologic response at 3 months CrCl 30–60 ml/min: 60% CrCl < 30 ml/min not on dialysis: 60% CrCl < 30 ml/min on dialysis: 20%	Grade 3/4 AEs 45% Most common AEs were anemia, decreased appetite, muscle weakness/fatigue Pneumonia: 19.3%
Phase II MM-013	Pomalidomide 4 mg days 1–21 Dex 40 mg weekly if <75 years of age or 20 mg weekly if >75 years of age	81 RRMM	eGFR < 45 ml/min/1.73 m^2^	HR ORR/VGPR or better A: 39.4%/18.2% B: 32.4%/8/8% C: 14.3%/7.1% Renal response A: 18.2%; complete RR in 18.2$% B: 35.3% C: 7.1%	Grade 3/4 AEs (A/B/C) Neutropenia - 60.6%/44.1%/57.1% Anemia - 27.3%/35.3%/57.1% Thrombocytopenia – 27.3%/17.6%/50% Infections – 39.4%/26.5%/28.6% Pneumonia – 12.1%/5.9%/7.1% Hyperkalemia – 0%/8.8%/14.3%

*AE* adverse event, *BDD and PAD* bortezomib doxorubicin dexamethasone, *CBD* cyclophosphamide bortezomib dexamethasone, *CR* complete response, *CrCl* – creatinine clearance by Cockcroft Gault method, dex dexamethasone, *eGFR* estimated glomerular filtration rate by Modification of Diet in Renal Disease (MDRD) method, Group A eGFR 30–<45 ml/min/1.73 m^2^, Group B eGFR < 30 ml/min/1.73 m^2^ not dialysis dependent, Group C dialysis dependent, *HR* hematologic response, *LCCN* light chain cast nephropathy, *MP* melphalan prednisone, *nCR* – near complete response, *NDMM* newly diagnosed multiple myeloma, *ORR* overall response rate, *PR* partial response, *RI* renal impairment (mild = CrCl ≥ 60 ml/min, moderate = ≥30 to <60 ml/min, severe =<30 ml/min), *RR* renal response, *RRMM* relapse or refractory multiple myeloma, *SAE* severe adverse event, *Scr* serum creatinine, *sRI* severe renal impairment with CrCl <30 ml/min, *VGPR* very good partial response, *VMP* bortezomib melphalan prednisone.

Limited data exist in the frontline therapy of severe renally impaired MM patients treated with the other proteasome inhibitors. A phase I/Ib study was conducted with ixazomib in severe renally impaired patients including 7 patients on dialysis [32]. A dose of 3 mg on Days 1, 8, 15 on a 28-days cycle was established for safety and pharmacokinetics standpoint but no efficacy data was provided. A study of carfilzomib in severe renally impaired patients including 10 patients on dialysis recommended the doses of 27 and 56 mg/m^2^ [33]. In a phase II trial of carfilzomib dexamethasone in renally impaired relapsed refractory (RRMM) patients using the 15 and 27 mg/m^2^ dosing, HR was similar in patients with varying degree of renal impairment including patients on dialysis [34]. They achieved an ORR of 25.5%, with all being PR or less. Adverse effects were also similar except for AKI in the 3 of 10 patients with moderate renal impairment (creatinine clearance between 30 and 49 ml/min) [34]. Unfortunately, the risk of renal toxicity including thrombotic microangiopathy makes carfilzomib an unattractive choice in MM patients with AKI [35, 36]. Currently, neither ixazomib nor carfilzomib is approved for frontline treatment of MM in the United States.

### Immunomodulators (Table 1)

Immunomodulators have been used in MM patients with renal impairment. Despite a similar mechanism of actions, they possess different pharmacologic properties that give each a unique pharmacokinetics and different side effects. Thalidomide is hydrolyzed by all body fluids and probably has limited renal clearance. Pharmacokinetic studies however showed clearance with dialysis, thus it should be given after dialysis [37]. Lenalidomide is renally cleared and dialyzable. It requires dose adjustment according to GFR and dialysis status [38]. Pomalidomide is renally secreted, but it is metabolized by the liver resulting in only mild extension of its half-life even in severe renal impairment making dosage adjustment for renal function unnecessary but should be given post dialysis [39].

There are only two small studies of thalidomide in renal failure patients with MM. The first is a 7 patient study where thalidomide was dosed between 100 mg and 400 mg daily in patients with creatinine clearance (CrCl) between 47 ml/min and dialysis dependence [40]. Three patients achieved a CR that lasted 5–8 months and 1 patient achieved a PR for 1 year. Adverse effects included constipation, peripheral neuropathy and hyperkalemia were noted in the dialysis dependent patient. In a separate study of 20 RRMM patients with renal impairment treated with thalidomide or thalidomide dexamethasone, 75% achieved a HR with 45% achieving a PR or better after stem cell transplant [41]. Dose reduction to 200 mg daily was required in 50% of patients treated with the 400 mg daily dose due to adverse effects.

Several phase II trials had been performed with lenalidomide dexamethasone (Rd) in renally impaired MM patients. The dosing schedule used was: 10 mg daily for CrCl >30 but ≤50 ml/min, 15 mg q48 hours for CrCl <30 ml/min but not on dialysis, and 5 mg daily after dialysis in patients on dialysis for 21 days on a 28-days cycle in one study [42]. In a small study of 35 NDMM and relapsed patients with high dose dexamethasone, 4 died during the first 2 cycles and 5 withdrew from the study, 3 were for adverse events. On an intention to treat analysis, 20% had a CR and 8% had a VGPR. Sixteen (45.7%) of the 35 patients had a renal response, with 14.2% achieving a renal CR, 11.4% with renal PR and 20% in renal MR. Another phase II trial of Rd using the same lenalidomide dosing but weekly dexamethasone achieved an ORR of 76% with 50% PR and 26% VGPR [43]. The ORR was similar between patients with normal kidney function and mild renal impairment vs patients with moderate renal impairment to ESRD; however, only 1 patient with severe renal impairment (eGFR < 30 ml/min/1.73 m^2^ or ESRD) achieved a VGPR. A dose escalation trial in renally impaired RRMM patients used a similar dosing schedule for dialysis independent patients dosed lenalidomide at 15 mg 3 times a week after dialysis for dialysis dependent patients [44]. The highest dosing achieved in the study was 25 mg daily for patients with CrCl 30–59 ml/min, 15 mg daily for those with CrCl < 30 ml/min regardless of dialysis status. The ORR was 54.3% with the poorest response seen in the dialysis group (20%). Twenty-eight patients experienced grade 3/4 adverse events with 1 death in a dialysis dependent patient from lung infection, sepsis and multiorgan failure attributed to therapy while 5 others died of unrelated deaths due to cirrhosis, intra-abdominal hemorrhage, sudden death and 2 ESRD. Median PFS was 12.6 months and OS was 20.0 months.

A phase II trial was conducted with pomalidomide and low dose dexamethasone in RRMM patients [45]. All 81 patients had an eGFR <45 ml/min/1.73 m^2^ with 14 dialysis dependent patients. Pomalidomide was dosed at 4 mg days 1–21 on a 28-days cycle. ORR was 39.4% in group A (patients with eGFR between 30 and 45 ml/min/1.73 m^2^), 32.4% in group B (patients with <30 ml/min/1.73 m^2^ not on dialysis) and 14.3% in group C (patients on dialysis). No CR was recorded but VGPR was noted in 18.2% of group A, 8.8% of group B and 7.1% of group C patients. Median PFS correlated with baseline kidney function [6.5 m (A) vs 4.2 m (B) vs 2.4 m (C)] and OS [16.4 m (A) vs 11.8 m (B) vs 5.2 m (C)] was the shortest among dialysis dependent patients. Leukopenia was more than twice as common in dialysis dependent patients but infection rates were similar. Thrombocytopenia was also most severe in the dialysis dependent patients. Dose reduction and discontinuation rates were similar amongst the 3 groups. Pomalidomide is currently approved for relapsed MM with 2 prior therapies including lenalidomide and a proteasome inhibitor.

### Anti-CD38 monoclonal antibodies

Currently, 3 randomized trials have been conducted with daratumumab in the upfront setting in MM. The addition of daratumumab to the backbone of Rd was compared in MAIA, bortezomib thalidomide dexamethasone (VTD) in CASSIOPEIA and bortezomib lenalidomide dexamethasone (VRD) in the GRIFFIN trial [46–48]. Unfortunately, all 3 trials excluded patients with an eGFR <30 ml/min/1.73 m^2^ so data on severe renal impairment or dialysis are limited to case reports for daratumumab [49]. A study comparing isatuximab, carfilzomib, dexamethasone vs carfilzomib plus dexamethasone has been conducted in RRMM with renal impairment (IKEMA trial) [50]. Unfortunately, in this study, only 2.7% and 2.4% of the patients had severe renal impairment.

### Extracorporeal therapies

Plasmapheresis (PLEX) for the treatment of AKI in MM was first reported in 1976 [51]. Since then, three randomized trials had been performed with differing outcomes. Zucchelli et al. randomized 15 patients to daily PLEX with hemodialysis vs 14 patients to peritoneal dialysis (PD) [52]. Patients who underwent PLEX had greater reduction of Bence Jones proteinuria than PD (*p* < 0.001). Eleven of 13 dialysis dependent patients treated with PLEX became dialysis independent but only 2 of 11 PD patients recovered kidney function. Although positive, this study was criticized for high early mortality (35.7%) in the PD group vs 6.7% in the PLEX treated patients. Johnson et al. randomized 21 patients to hemodialysis vs hemodialysis plus thrice weekly PLEX. Dialysis was required for 7 of 11 PLEX and 5 of 10 hemodialysis patients [53]. Kidney function improved in 63.6% of the PLEX patients vs 50% of the hemodialysis patients (*p* = NS), but of the dialysis dependent patients, all 3 who recovered had received PLEX. Clark et al. randomized 58 patients to 5–7 PLEX over the first 10 days and 39 patients to the control group. Hemodialysis was required for 25.9% of the PLEX patients vs 36% of the controls [54]. At the end of the study, dialysis requirement and death were noted in 17.9% and 33.3% of the controls and 8.6% and 32.8% of the PLEX patients respectively, (p = NS). The primary endpoint (a composite of death, dialysis dependence and eGFR < 29 ml/min/1.73 m^2^) was documented in 57.9% of PLEX patients vs 69.2% of the controls, *p* = 0.36.

Although some felt that PLEX was ineffective based on the Clark study, it is important to point out significant differences among these studies. LCCN was confirmed by kidney biopsy in majority of the patients in the Zucchelli and Johnson studies, but few biopsies were performed in the Clark study [52–54]. Dialysis requirement was the threefold higher in positive Zucchelli study than the negative Clark study. Investigators in the Johnson study also felt that PLEX was more beneficial in patients with the more severe AKI. Finally, patients in the Zucchelli study received daily PLEX while the Johnson and Clark studies were limited to every other day [52–54].

Other extracorporeal devices used in the treatment of LCCN includes the high cut-off (HCO) dialyzers. HCO dialyzers are dialyzers with pore size up to 50 kd (vs 5 kd in normal dialyzers) which can reduce serum FLC levels by >70% [55]. Two randomized trials have been performed to date. MYRE was the first trial to report enrolled 98 dialysis dependent patients in France from 2011 to 2016 [23]. Patient were treated with BD on a 21-day cycle and cyclophosphamide could be added after cycle 3 if response was insufficient. Eight 5 h HCO dialysis were performed within the first 10 days while the control group received the same with a regular dialyzer. HCO dialyzer treatment produced a higher ORR (78.3% vs 60.4%, p 0.06) and significantly higher >VGPR rate (69.6% vs 47.9% respectively, OR - 2.37, *p* = 0.03) as compared to control. HCO hemodialysis resulted in a significantly higher renal recovery at 6 months (secondary endpoint, 56.4% vs 35.4% respectively, odds ratio – 2.37, *p* = 0.04) and 12 months (60.9% of the HCO vs 37.5% of control, OR 2.59, *p* = 0.02) but did not reach significance at 3 months (primary endpoint, 41.3% vs 33.3% respectively, *p* = 0.42). There was no difference in OS.

The EuLITE study enrolled 90 dialysis dependent patients mainly in the United Kingdom from 2008 to 2013 used BDD as the chemotherapy [56]. The HCO dialysis was scheduled for 6 h the first day followed by another seven 8 h sessions over 10 days. Control patients received 4 h dialysis thrice weekly. Despite similar single session FLC reductions (-77% for κ, -72% for λ) as MYRE, patients in EuLITE treated with the HCO dialyzer had a lower CR (14% vs 30%) and VGPR (23% vs 32%) at 6 months as compared to control patients [23, 56]. Moreover, the HCO group had an inferior age adjusted OS (hazard ratio – 2.63, *p* = 0.03) as compared to control despite similar renal recovery rates at 90 days (56% HCO vs 51% control, p - 0.81) [56]. Higher infectious complications especially pulmonary infection in the HCO group vs control (31% vs 9% respectively) may have played a role.

### Recommendations

**Fig. 1:**
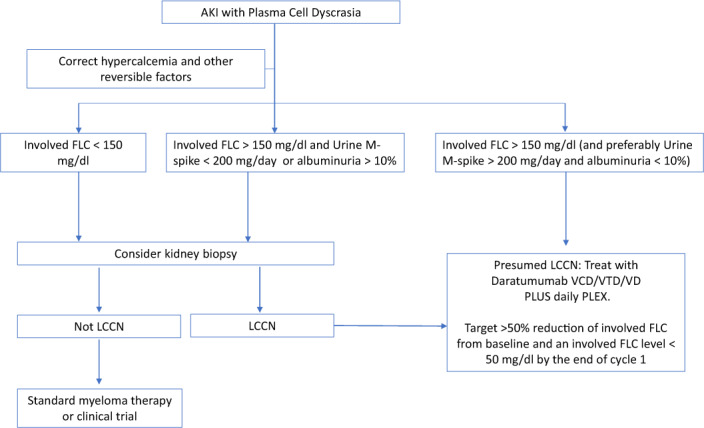
Treatment algorithm for newly diagnosed multiple myeloma patients with acute kidney injury. AKI acute kidney injury, FLC free light chains, M monoclonal, LCCN light chain cast nephropathy, VCD bortezomib, cyclophosphamide, dexamethasone, VTD bortezomib, thalidomide, dexamethasone, PLEX plasma exchange.

Given the importance of renal recovery and the requirement for rapid serum FLC reduction, an aggressive therapeutic approach is justified in patients MM patients with AKI. Regimens used should have a high and rapid response rate; the drugs used should not require modifications for renal function and should be readily available for immediate administration. The goal should be to reduce circulating serum FLCs as quickly as possible, including the use of PLEX (Fig. 1). In NDMM, based on the activity of various drugs and treatment regimens used as initial therapy so far, we prefer daratumumab combined with VCD (bortezomib cyclophosphamide dexamethasone) or VD as initial therapy. We have found high activity with VCD/VD in the past [57], but in our clinical experience and trial data from Andromeda [58], the addition of daratumumab hastens the response and limits the number of days extracorporeal light chain removal that is needed. In countries where IMiDs can be easily obtained for hospitalized patients, daratumumab plus VTD such as the regimen used in CASSIOPEIA could be an option instead of daratumumab plus VCD [48]. Lenalidomide should probably be avoid in the upfront setting due to requirement for dose adjustment.

Extracorporeal light chain removal with PLEX should started as soon as possible to help reduce the serum FLC concentration more rapidly. Even though data on PLEX is controversial, the procedure has low risk, and in our opinion provides patients with the best chance at renal recovery [20, 57]. PLEX should be performed daily until the involved FLC is below 150 mg/dl or >60% reduction from baseline, if possible. Since PLEX could potentially remove daratumumab, daratumumab should be given after PLEX. In countries where HCO dialyzers are available, these could be an option instead of PLEX, although the 5 h sessions used in the MYRE trial should be performed.

The situation is more complicated with RRMM patients since many of the relapsed medications have not been tested in severe renal impaired patients. In these patients, renal recovery is equally important since eligibility of CAR-T cell therapy and clinical trials require an eGFR >40–50 ml/min/1.73 m^2^. If the patient did not relapse on an anti-CD38 antibody drug, daratumumab or isatuximab could be used. Combination therapy based on bortezomib, dexamethasone, thalidomide and a 4-day continuous infusion of cisplatin, doxorubicin, cyclophosphamide, and etoposide (VDT-PACE) can be used in renally impaired patients. In these patients, cisplatin is usually omitted and cyclophosphamide should be dose adjusted for kidney function. PLEX should be offered especially in patients with options of potential future therapies.

## Data Availability

This is a current treatment algorithm. There are no new data generated for this paper and data sharing is not applicable.
